# Experimental warming increases herbivory by leaf‐chewing insects in an alpine plant community

**DOI:** 10.1002/ece3.2398

**Published:** 2016-09-07

**Authors:** Tone Birkemoe, Saskia Bergmann, Toril E. Hasle, Kari Klanderud

**Affiliations:** ^1^ Department of Ecology and Natural Resource Management Norwegian University of Life Sciences P.O. Box 5003 N‐1432 Ås Norway

**Keywords:** Alpine, biotic interactions, *Bistorta vivipara*, climate change, *Dryas octopetala*, insect herbivory

## Abstract

Climate warming is predicted to affect species and trophic interactions worldwide, and alpine ecosystems are expected to be especially sensitive to changes. In this study, we used two ongoing climate warming (open‐top chambers) experiments at Finse, southern Norway, to examine whether warming had an effect on herbivory by leaf‐chewing insects in an alpine *Dryas* heath community. We recorded feeding marks on the most common vascular plant species in warmed and control plots at two experimental sites at different elevations and carried out a brief inventory of insect herbivores. Experimental warming increased herbivory on *Dryas octopetala* and *Bistorta vivipara. Dryas octopetala* also experienced increased herbivory at the lower and warmer site, indicating an overall positive effect of warming, whereas *B. vivipara* experienced an increased herbivory at the colder and higher site indicating a mixed effect of warming. The Lepidoptera *Zygaena exulans* and *Sympistis nigrita* were the two most common leaf‐chewing insects in the *Dryas* heath. Based on the observed patterns of herbivory, the insects life cycles and feeding preferences, we argue that *Z. exulans* is the most important herbivore on *B. vivipara,* and *S. nigrita* the most important herbivore on *D. octopetala*. We conclude that if the degree of insect herbivory increases in a warmer world, as suggested by this study and others, complex interactions between plants, insects, and site‐specific conditions make it hard to predict overall effects on plant communities.

## Introduction

Global warming is particularly pronounced in northern areas, and mean temperatures are predicted to increase by 0.3–4.8°C by year 2100 (IPCC, 2013). This is already affecting species and trophic interactions worldwide (Parmesan and Yohe 2003; Parmesan 2006; Tylianakis et al. 2008; Walther 2010). Arctic and alpine ecosystems are expected to be especially sensitive to climate change (Parmesan 2006), with major shifts in biodiversity and species composition (Sala et al. 2000).

Most research on climate warming effects in arctic and alpine regions has focused on plants (Arft et al. 1999; Walker et al. 2006; Pieper et al. 2011; Elmendorf et al. 2012a,b). Many species have expanded their distributions to higher altitudes and latitudes (Klanderud and Birks 2003; Pauli et al. 2012; Grytnes et al. 2014), and plant phenology is changing with increasing temperature (Oberbauer et al. 2013). Some functional groups, such as shrubs and graminoids, are increasing in abundance in many areas, whereas others, such as dwarf shrubs, are decreasing (Klanderud and Totland 2005; Walker et al. 2006; Elmendorf et al. 2012a,b). Interestingly, responses within and between different groups of species vary across regions and are not always directly related to changes in temperature (Elmendorf et al. 2012a; Grytnes et al. 2014). Reasons for this inconsistency are suggested to result from interactions with other driving factors, such as site‐specific conditions of snow, soil moisture, or herbivore pressure (Elmendorf et al. 2012a,b; Grytnes et al. 2014).

Even though insect–plant interactions are important in order to understand how alpine and arctic ecosystems respond to climate warming (Roy et al. 2004; Pedersen and Post 2008; Tylianakis et al. 2008; Liu et al. 2011; Gillespie et al. 2013), surprisingly, few studies have investigated this trophic interaction in light of climate change (but see Richardson et al. 2002; Roy et al. 2004; Høye et al. 2013; Barrio et al. 2016). The general prediction is an increase in herbivory with increasing temperature (Tylianakis et al. 2008), but the few experiments carried out have found contrasting results; herbivory may increase, decrease, or remain unchanged (e.g., Richardson et al. 2002; Roy et al. 2004; Liu et al. 2011; Gillespie et al. 2013). Several mechanisms may contribute to these diverging results. First, insects and host plant phenology may or may not change synchronously. With asynchronous changes, the herbivores can be forced to change host plants, increasing herbivory on new hosts and reducing herbivory on old (Liu et al. 2011). Second, responses in leaf nutrients, growth, and defensive compounds depend on the specific plant species (Richardson et al. 2002; Nybakken et al., 2008; Barrio et al. 2016). Thus, herbivore food quality may change inconsistently across sites, affecting herbivore food preferences and population growth differently. Third, insects’ feeding activity depends on their thermal adaptation (Liu et al. 2011; Barrio et al. 2016). Thus, whereas some species will increase their overall consumption and experience population growth, some may suffer from heat stress and a possible population decline. Finally, whereas arctic and alpine areas have been defined as simple ecosystems, a multitude of interactions occur (Wirta et al. 2015) which may further modify insect–plant interactions in a changing climate.

In order to understand plant community changes in alpine and tundra ecosystems in response to warming, studies of insect–plant interactions are essential. At present, very few such investigations have been carried out and it is difficult to generalize the responses. In this study, we test the effect of warming on herbivory in an alpine *Dryas* heath at Finse, southern Norway, in order to enable future predictions. Long‐term experiments are particularly valuable to understand climate warming effects on complex interactions (e.g., Adler et al. 2007), and we use two ongoing warming experiments (open‐top chambers, OTCs) at two elevations. We test whether long‐termed warmed plants are more affected by leaf‐chewing insects than control plants in ambient temperature by recording feeding marks inside and outside OTCs, and whether this effect differs between two sites at different elevations. To relate insect herbivores to the observed patterns of herbivory, we carried out a small inventory identifying the most important leaf‐chewing insects within the two sites. Our overall expectations were that insect herbivory would increase with experimental warming and be highest in the warmer site at the lowest elevation. However, we also expected the results to vary with plant and insect species present.

## Materials and Methods

### Study area and experimental setup

The study was conducted at Mount Sandalsnuten at Finse (60°36′ N, 7°31′ E) in southwestern Norway. The climate is alpine‐oceanic with an annual average temperature of −2.1°C and precipitation of 1030 mm (Moen 1998; The Norwegian Meteorological Institute 2015). The project was carried out from June to August 2012, a period with a mean monthly temperature of 6.3°C (Aune 1993) and 89 mm precipitation (at 1224 m a.s.l.) (Førland 1993).

The two experimental sites, defined as low site (leeside, ca. 1450 m a.s.l.) and high site (ridge, ca. 1550 m a.s.l.), are both situated in *Dryas* heaths. These are species‐rich plant communities (ca 10 vascular species per 50 × 50 cm plot) dominated by *Dryas octopetala,* and with other common species, such as *Bistorta vivipara, Saussurea alpina, Salix reticulata, Carex rupestris,* and *C. vaginata*. The high site is more exposed to wind, so the snow melts earlier and the growing season is approximately 3 weeks longer (Nybakken et al. 2011). The mean air summer temperature (July and August) is ca 0.8°C higher in the low than the high site (8.7°C vs. 7.5°C), whereas mean soil temperature (ca. 5 cm below ground) is 0.3°C higher in the low site (7.5°C vs. 7.2°C) (Nybakken et al. 2011).

Open‐top chambers (OTCs) are a common method to study global warming experimentally and are considered to have few undesired side effects (Marion et al. 1997; Arft et al. 1999; Hollister and Webber 2000). The low site experiment was set up in 2003 (Sandvik and Eide 2009) and the high site in 2000 (Klanderud and Totland 2007). The OTCs increase mean air temperature 5 cm above ground level by ca. 1.5°C and soil temperature by ca. 1.0°C in both sites (Klanderud and Totland 2005; Sandvik and Eide 2009). This is in accordance with an expected warming of 0.2–0.5°C per decade until 2050 for the Norwegian mainland (Hanssen‐Bauer and Førland 2001). Due to small‐scale topography, there are gaps of several cm at many points between the OTCs and the ground, and insects as well as rodents may move more or less freely in and out (see also Richardson et al. 2002; Barrio et al. 2016). We also observed *Zygaena exulans* larvae crawling on the OTC walls, and they were clearly not limited by the barrier. As for adult insects, they can fly freely in and out the ca 60‐cm open tops of the OTCs. The insects do not live their entire life within the OTCs as do plants. Therefore, we are not able to simulate the full effect of climate change, that is, simultaneous change in insect and plant phenology and dynamics. However, choosing to stay in plots of increased temperature will speed up insect development relative to the colder ambient conditions and it is likely to have a measureable effect on insect life cycles. However, the main effect that we are targeting with the experimental heating is the changes in feeding capacity. However, by simultaneously comparing sites of high and low elevation, we add effects of population dynamics and phenology that are likely to vary between these sites.

In this study, 10 control plots and 10 OTCs with similar plant composition were used at each site. Inner diameter at ground level of the OTCs is ca 0.9 m (low site) and 1 m (high site). Distance between OTCs and control plots is ≥1 m (low site) and ≥2 m (high site).

To detect differences in insect herbivory, we recorded feeding marks of leaf‐chewing insects on leaves of all vascular plant species in all the 40 plots in early (19th to 28th of June) and late (28th of July to 4th of August) summer. The data were collected within a 50 × 50 cm frame placed in the middle of the OTCs and control plots. The frame was divided into 10 × 10 cm subplots to facilitate data collection.

In addition to the 50 × 50 cm frame, we searched the whole inside of the OTCs and an additional 15‐cm zone at all sides of the control plots for 15 min and recorded and took pictures of all leaf‐chewing insects observed before analyzing the vegetation for feeding marks. In order not to disturb the experiment, no insects were removed from the plots. Therefore, species identification was performed at the sites or later based on photographs. We recorded feeding marks on all living vascular plants inside the frame in each plot and the amount (%) of each leaf removed. These two estimates represent the total feeding activity of herbivores (number of feeding marks) and to what extend the herbivores utilize each leaf (% of each leaf removed).

The percent cover of plant species with most feeding marks (*D. octopetala, B. vivipara, and S. reticulata*) was estimated inside the frame at the low site in early and late summer (no significant difference with season). For the high site, we used vegetation data recorded in the late season in 2011 (Olsen and Klanderud 2013). The percentage cover of *D. octopetala* varied from 4.8 to 80.0 (mean 21.4) at the low site and 5.0–95.0 (mean 54.7) at the high site. As for *B. vivipara*, the cover varied from 0.5% to 22.5% (mean 4.4) at the low site and 1% to 4% (mean 2.1) at the high site.

### Insect trapping

To get a rough estimate of insect herbivores present at the two sites, we used five pitfall traps (6 cm in diameter, 10 cm deep) filled half way up with propylene glycol and water (proportion 8:2, respectively) and a few drops of soap. The traps were protected by a plexiglass roof and mounted in the *Dryas* heath more than 10 m away from the experimental plots at both sites the 20th of June. The traps were emptied every 2 weeks, 3rd of July (early summer), 28th of July (mid‐summer), and 11th of August (late summer). Data from the five pitfall traps were pooled within each site to minimize workload. The insects were preserved in 70% alcohol, and the leaf‐chewing species were identified in the laboratory to the lowest taxonomic level possible.

### Statistical analysis

*Dryas octopetala* and *B. vivipara* were the only species with enough feeding marks for analyzing statistically, and we ran models for these two species separately. To test the effect of site (low, high) and treatment (control, OTC) on the amount of insect herbivory, we used an herbivory index defined as “number of feeding marks divided by percentage cover of the plant fed on” as a response variable in a factorial ANOVA analysis. We also did the analysis with number of feeding marks as a response variable and percent cover of the given plant species as a covariate in an ANCOVA analysis, which gave the same overall results as the index (Table S1, Figs. S1, S2). We also tested whether site and treatment effected the amount (%) of each leaf eaten (response variable) with an ANCOVA analysis, including percentage cover of the particular plant species added as a covariate. Knowing that the experiments had been running for several years and might have affected the plant species cover, we also included the interaction term between cover of the particular species and treatment.

The two recordings of feeding marks in time (early and late summer) are highly dependent and could not be included in the same analysis. Thus, linear models were performed with data from early and late summer separately. The response variables were log‐transformed to reach normality if needed.

All analyses were carried out in JMP version 11.1.1. (2013 SAS Institute Inc, Cary, NC 27513‐2414).

## Results

As many as 94% of 4744 feeding marks recorded were found on two plant species, *Dryas octopetala* (53%) and *Bistorta vivipara* (41%). The remaining marks were found on *S. reticulata*,* Salix herbacea*,* Vaccinium uliginosum, Saussurea alpina*,* Parnassia palustris*,* Poa alpina*,* Ranunculus acris*,* Thalictrum alpinum*,* Tofieldia pusilla,* and *Carex* sp*.,* but were too few to allow for statistical analysis.

**Figure 1:**
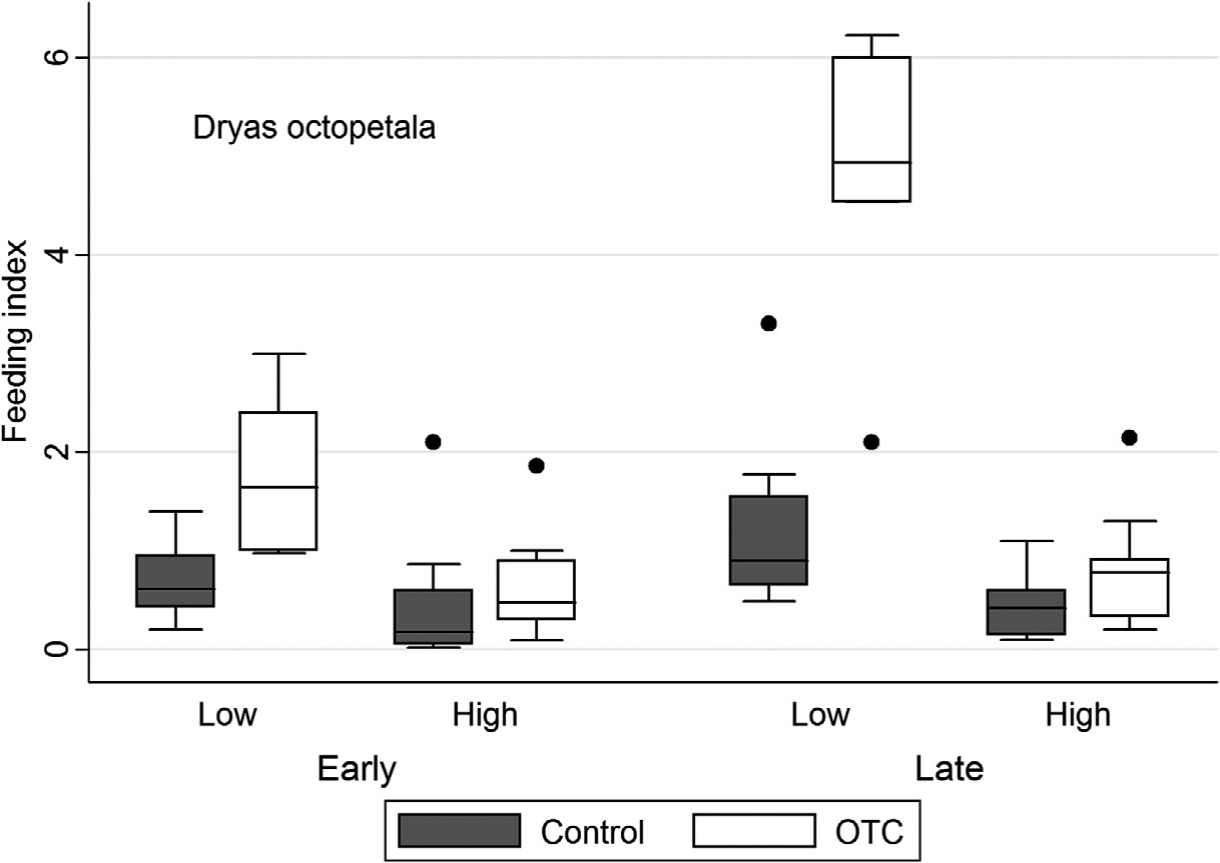
Herbivory on *Dryas octopetala* in control and open‐top chamber plots expressed as a feeding index (number of feeding marks/%cover of *D. octopetala*) at a low and a high‐elevation site recorded in early and late summer in an alpine *Dryas* heath at Finse, Norway.

**Figure 2:**
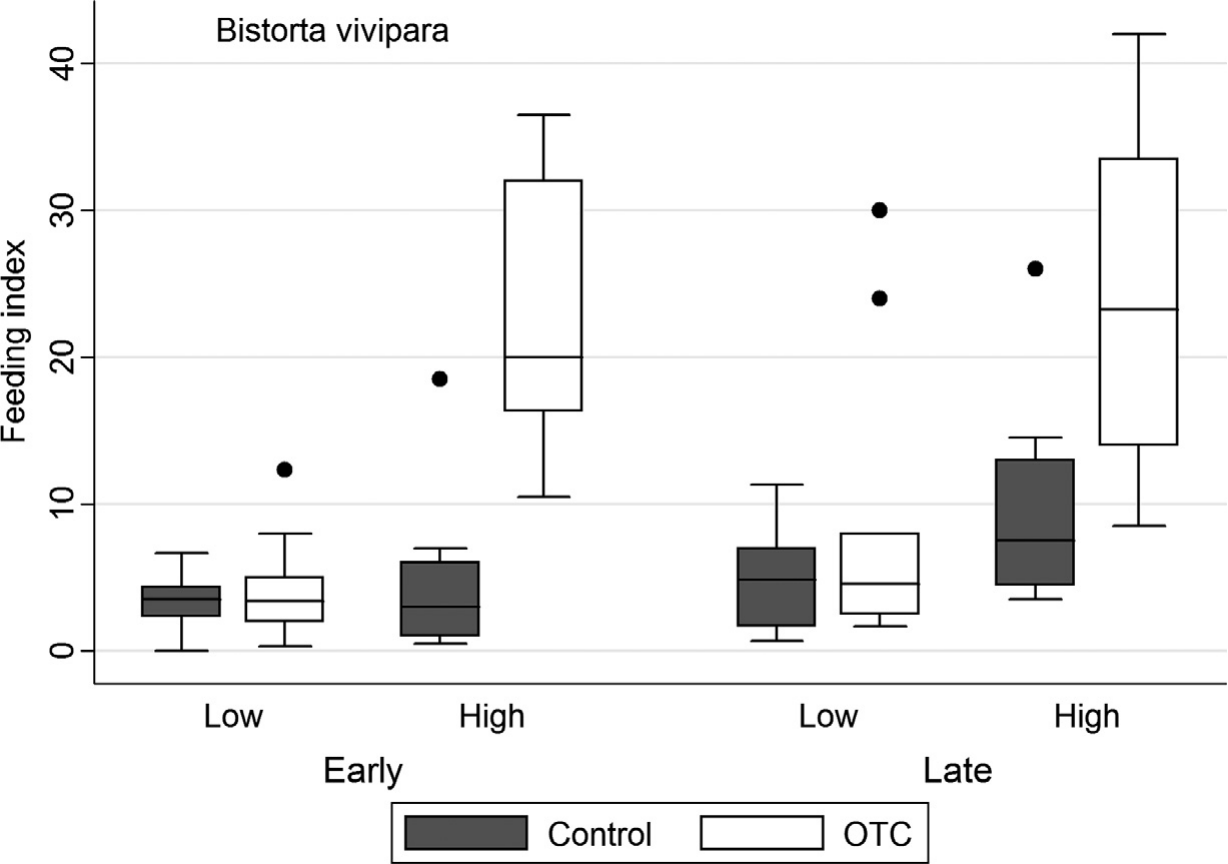
Herbivory on *Bistorta vivipara* in control and open‐top chamber plots expressed as a feeding index (number of feeding marks/%cover of *B. vivipara*) at a high and a low‐elevation site recorded in early and late summer in an alpine *Dryas* heath at Finse, Norway.

Experimental warming increased feeding on *D. octopetala* mainly at the low site in late summer (Table 1, Fig. 1). In contrast, herbivory on *B. vivipara* increased with experimental warming mainly at the high site in early summer (Table 1, Fig. 2). Feeding on *D. octopetala* was overall higher at the lower site, whereas herbivory on *B. vivipara* was overall higher at the high site. The relative herbivory on *B. vivipara* was many orders of magnitude higher than the herbivory on *D. octopetala* (Figs. 1, 2).

**Table 1:** ANOVA analysis with feeding index (number of feeding marks/% cover of species) on *Dryas octopetala* and *Bistorta vivipara* as response variables explained by site, treatment and the interaction term

	Early summer			Late summer	
	df	*t*‐ratio	*P*‐value	*t*‐ratio	*P*‐value
*Dryas octopetala*					
Site (Low–High)	1	**3.74**	**0.0007**	**6.69**	**<0.0001**
Treatment (OTC–Control)	1	−**2.99**	**0.0052**	−**4.62**	**<0.0001**
Site × Treatment	1	−0.05	0.9640	−**2.18**	**0.0368**
	*n* = 37, *R* ^2^ = 0.40			*n* = 37, *R* ^2^ = 0.67	
*Bistorta vivipara*					
Site (Low–High)	1	−**3.25**	**0.0025**	−**4.35**	**0.0001**
Treatment (OTC–Control)	1	−**3.24**	**0.0026**	−**2.93**	**0.0058**
Site × Treatment	1	**4.17**	**0.0002**	1.07	0.2906
	*n* = 39, *R* ^2^ = 0.53			*n* = 40, *R* ^2^ = 0.44	

Data from early and late summer are analyzed separately. Significant (*P* < 0.05) effects are in bold. All response variables were log‐transformed prior to analysis.

**Figure 3:**
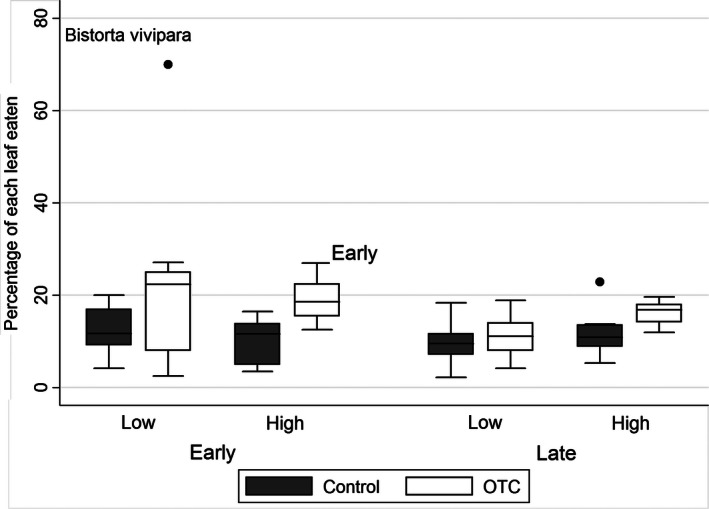
Percentage of each *Bistorta vivipara* leaf eaten by insect herbivores in control and open‐top chamber plots at a low and a high‐elevation site in an alpine Dryas heath at Finse, Norway.

In addition to the increased number of feeding marks, the insect herbivores also consumed more of each *B. vivipara* leaf when warmed experimentally. This effect was apparent in early and late summer (Table 2, Fig. 3). The consumption of each *D. octopetala* leaf did not increase with experimental warming (Table 2).

**Table 2:** ANCOVA results with mean percent of each *Dryas octopetala* or *Bistorta vivipara* leaf removed by insect herbivores per plot as response variables explained by site, treatment, and the percent cover of each of these plant species within the plots

	Early summer			Late summer	
	df	t‐ratio	*P*‐value	t‐ratio	*P*‐value
*Dryas octopetala*					
Site (Low–High)	1	1.49	0.1468	−0.04	0.9661
Treatment (Control–OTC)	1	−0.21	0.8342	−1.41	0.1699
Site × Treatment	1	−0.44	0.6640	0.39	0.7023
% *Dryas*	1	0.37	0.7154	0.54	0.5916
% *Dryas* × Treatment	1	0.05	0.6183	−0.88	0.3858
	*n* = 37, *R* ^2^ = 0.10			*n* = 37, *R* ^2^ = 0.13	
*Bistorta vivipara*					
Site (Low–High)	1	0.16	0.8744	−1.93	0.0614
Treatment (Control–OTC)	1	−**2.55**	**0.0158**	−**2.29**	**0.0284**
Site × Treatment	1	0.91	0.3719	1.10	0.2786
% *Bistorta*	1	0.13	0.8936	−0.74	0.4656
% *Bistorta* × Treatment	1	−0.55	0.5854	−0.46	0.6477
	*n* = 39, *R* ^2^ = 0.19			*n* = 40, *R* ^2^ = 0.27	

Significant (*P* < 0.05) effects are in bold. Percent leaf removed was log‐transformed in the analysis of *B. vivipara* in early summer.

### Insect species

Lepidoptera larvae were the only leaf‐chewing insect herbivores found that may feed on vascular plants (Table 3). *Zygaena exulans* (Hohenwarth 1792) comprised 63.4% of the 41 Lepidoptera larvae found in the high site and 62.5% of the 24 larvae found in the low site. The comparable proportion was 12.2% and 20.8% for *Sympistis nigrita* (Boisduval 1840) in the high and low site, respectively. The other Lepidoptera larvae were a mix of several species but only *Erebia pandrose* (Borkhausen, 1798) (one observation at low site in late summer) was identified to species. The pitfall traps and the observations in the control and OTC plots gave similar information of the leaf‐chewing insect fauna: *Z. exulans* were present in the first part of the summer, whereas *S. nigrita* and the other unidentified larvae were more common in the later part (Table 3). *Zygaena exulans* were restricted to early summer only in the lower site, whereas the occurrence was extended to mid‐ and late summer at the high site.

**Table 3:**
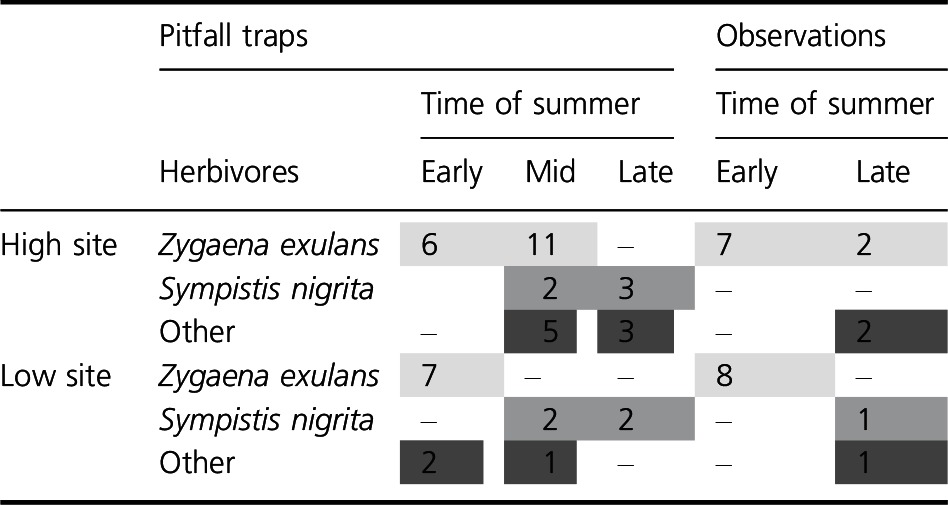
Lepidoptera larvae caught in five pitfall traps or observed in control and open‐top chamber plots at the low and high‐elevation sites at Mt. Sandalsnuten, Finse, Norway. The shading indicates occurrence of different species or species groups in time

## Discussion

We have shown that both *Dryas octopetala* and *Bistorta vivipara* experienced increased insect herbivory with experimental warming. These results are in line with our expectations and with several other findings at ecological as well as evolutionary scales (Wilf and Labandeira 1999; Roy et al. 2004; Kozlov 2008; Liu et al. 2011; Gillespie et al. 2013; Lemoine et al. 2013). However, we also expected more herbivory at the lower (warmer) than the higher (colder) site as they differed by almost 1°C during the summer months. This pattern was found for *D.octopetala*, giving the highest experienced herbivory in the warmest (lowest) experimentally heated plots. Herbivory on *B. vivipara,* on the other hand, showed the opposite pattern with reduced herbivory in the lower (and warmer) plots relative to the higher (and colder) plots, and the response to experimental warming was mainly present at the upper and colder site.

Several mechanisms might account for the unexpected increased herbivory on *B. vivipara* at the colder site. First, timing of snowmelt has been identified as an important factor for insect phenology and activity in the arctic (Høye et al. 2013, 2014) and day of snowmelt may therefore be the main driver of herbivory rates (Roy et al. 2004). In our study area, the vegetation melts out about 3 weeks earlier at the high than the low site (Nybakken et al. 2011). Thus, increased herbivory at the high and cold site for *B. vivipara* might be explained by early snowmelt speeding up local insect phenology. Second, a lower consumption of the favorite food (*Salix*) of the caterpillar *Gynaephora groenlandica* was found in the lowest (and warmest) sites in Canada, with the highest herbivory at intermediate (medium warm) sites (Barrio et al. 2016). This was explained by a narrow thermal adaptation of this highly arctic species, not enabling it to increase in response to heat in the lower (warmer) range of its distribution. A similar mechanism may also prevent increased feeding in the lower (warmer) site in our system. Although the rise in temperature inside the OTCs relative to the ambient temperatures at our high‐elevation site is larger than the relative change from the high to the low site, the insects larvae within the OTCs may move out to cool down if needed. At the lower site, however, this is not an option and heat stress might be more severe, restricting feeding at increased temperatures.

If different herbivores are responsible for the feeding marks on the two different plant species, the mechanisms described above may be species dependent, creating the contrasting patterns between *D. octopetala* and *B. vivipara* in our study. The caterpillar *Sympistis nigrita* is monophagous on *D. octopetala* and was the most important herbivore registered on this plant species at northeastern Greenland (Roslin et al. 2013). Larvae of *S. nigrita* were the second most abundant insect herbivore found at our sites at Finse. *Sympistis nigrita* is known to hatch from eggs in early spring and pupate in July–August after 3–4 weeks of feeding (Roslin et al. 2013). The youngest larval instar feeds on flowrs, preferring pistils and stamens (Roslin et al. 2013). As it develops, and *Dryas* flowers senesce, it changes the diet toward the leaves (Hopkins 2012). This fits well with our results of increased herbivory on leaves in late summer only. Adding to this pattern, we also found *S. nigrita* larvae to be active in the later part of summer (Table 1). As warming generally increases abundance of flowers in *Dryas* (personal observations, Welker et al. 1997), increased feeding of *S. nigrita* is indeed expected. *Sympistis nigrita* was found close to both sites, but we did not estimate abundances properly in the plots. However, given that the feeding marks on *D. octopetala* actually represent *S. nigrita*, the raised levels of herbivory in the warmer relative to the colder site may reflect a population response in *S. nigrita* due to increased food availability.

Whereas the feeding marks on *D. octopetala* match the seasonal occurrence of *S. nigrita*, the feeding marks on *B. vivipara* may be explained by the activity of the highly polyphagous *Z. exulans,* having *B. vivipara* as one of many food plants (Naumann et al. 1999). These butterfly larvae are likely to have a 2‐year life cycle at Finse (Hågvar, 1976) as relatively large larvae occur as soon as the snow disappears. The larvae terminate feeding very soon if kept at higher temperatures (Hågvar, 1976; Hasle 2013). In addition, we found a slightly longer activity period in the high (colder) than in the low (warmer) site. These observations combined suggest a similar adaptation in the alpine *Z. exulans* as indicated in the arctic *G. groenlandica*, an inability to use temperatures above a certain threshold (Kukal and Dawson 1989; Barrio et al. 2016). Thus, when the snow melts out at the lower site, the temperature may already be too high for *Z. exulans*, not promoting feeding inside the OTCs. Parasitoids were frequently observed on *Z. exulans* in the field and hatched from paralyzed larvae when transferred to laboratory (unpublished results). Parasitism causing more than 50% mortality has been suggested to drive the phenology of *G. groenlandica* (Kukal and Kevan 1987), and the narrow temperature range seen in those two species might be linked to avoidance of predation rather than a pure thermal adaptation. Whether the increased effect of herbivory on the high relative to the low site is related to consumption rates or differences in population densities has to be investigated further.

More of each *B. vivipara* leaf was consumed by insects when experimentally warmed, whereas the percentage of each *D. octopetala* leaf removed remained unchanged. Assuming that the feeding marks on the two plant species were caused by different herbivores, species‐specific feeding pattern in response to temperature may explain this discrepancy. Following the line of thought from the experiments with *G. groenlandica*, heat stress changed the preferred food plant from *Dryas* to the more nutritious *Salix* when warmed (Barrio et al. 2016). Here, we suspect *Z. exulans* to feed on *B. vivipara* which is a highly nutritious plant. However, if stressed by heat, costs of movements between the patchily distributed *B. vivipara* plants might have increased and promoted longer feeding time within patches (plants) (Kamil, Krebs and Pulliam 1987). As for herbivory on *Dryas*, the herbivory index showed an increasing trend with warming across sites and heat stress is unlikely to be important. Also, *Dryas* form large mats which indicate very low movements costs between leaves. Dryas is also protected by high concentrations of defensive compounds (Nybakken et al., 2011), which may interfere with insect feeding patterns. *Dryas octopetala* respond to warming by producing larger and heavier leaves (Nybakken et al., 2008; Barrio et al. 2016). As we used the percentage of each leaf eaten to estimate herbivore consumption, an increase in leaf size might actually counteract effect. Thus, in later studies, amount of removed tissue should be used to estimate leaf consumption rather than a proportion of each leaf.

The larvae of *Z. exulans* are considered a polyphagous herbivore (Naumann et al. 1999), feeding on many host plants from different families. Hågvar (1976) found *Z. exulans* to feed mainly on *Salix* at lower elevations at Finse, but feeding marks on *Salix* were few in the present study. Generalist herbivores often specialize on the most abundant plant groups (Wilf and Labandeira 1999). Based on this argument, both *D. octopetala* and *B. vivipara* are likely to be preferred plants at our sites by any generalist herbivore*. Z. exulans* is known to feed on *B. vivipara* (Naumann et al. 1999) and was able to feed on *Dryas* in a no‐choice feeding experiment in the laboratory (Hasle 2013). Thus, *Z. exulans* and other generalist herbivores not identified (Table 3) might potentially feed on both plant species, complicating the overall picture in our study.

Our results are in line with recent meta‐analyses on plant community responses to climate change, suggesting that site‐specific conditions, such as ambient temperature, herbivory, moisture, and snow patterns, are important drivers of community dynamics in addition to climate warming. Although we find a general increase in herbivory, we also show complex interactions between plants and insects, possibly linked to phenology and species‐specific food preferences. Herbivory on *Dryas*, most likely caused by the monophagous *S. nigrita* feeding primarily on flowers, is likely to increase in importance in a warmer future and might already contribute to *Dryas* heath community dynamics by reducing Dryas seed production. Herbivory on *B. vivipara* is likely to be most important in the cooler sites and possibly deceases in importance with increasing temperatures. Clearly, the outcome of plant–insect interactions on plant community level in a warmer world is hard to predict, and more studies confirming possible outcomes are still needed.

## Conflict of Interest

None declared.

## Supporting information


**Figure S1.** Number of feeding marks on *D. octopetala* in control and OTC plots at a low and a high elevation site recorded in early and late summer in an alpine Dryas heath at Finse, Norway.

**Figure S2.** Number of feeding marks on *B. vivipara* in control and OTC plots at a high and a low elevation site recorded in early and late summer in an alpine Dryas heath at Finse, Norway.

**Table S1.** ANCOVA results with number of feeding marks on *D. octopetala* and *B. vivipara* as response variables explained by site, treatment and the percentage cover of these species within the plots.
