# Supplementary figures and images for: Experimental warming increases herbivory by leaf‐chewing insects in an alpine plant community
Source: Ecol Evol. 2016 Sep 7;6(19):6955–62. doi: 10.1002/ece3.2398 (PMC5513215; doi:10.1002/ece3.2398)

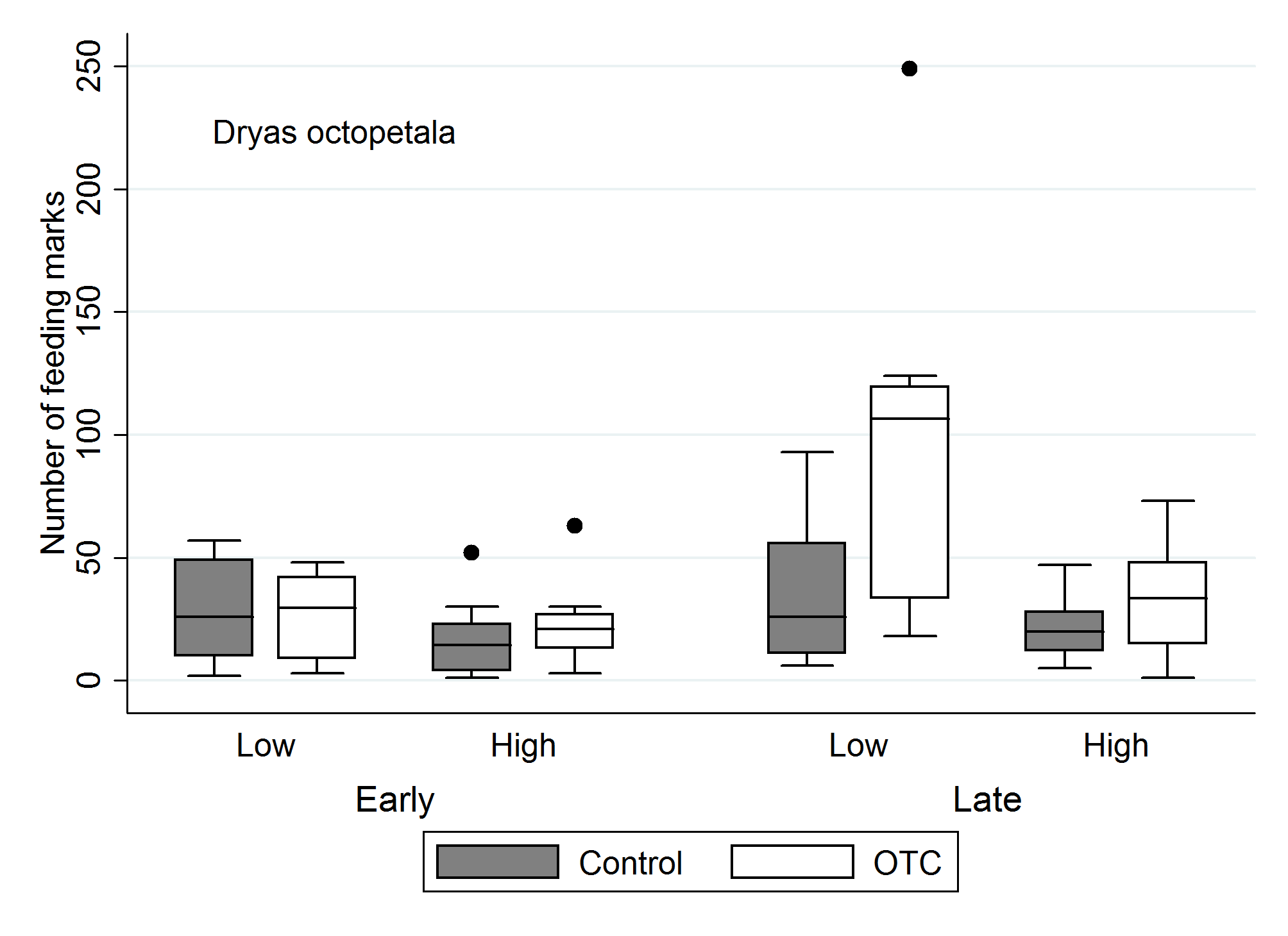

Supplement: Supplementary file 1 — Figure S1. Number of feeding marks on D. octopetala in control and OTC plots at a low and a high elevation site recorded in early and late summer in an alpine Dryas heath at Finse, Norway. [file ECE3-6-6955-s001.tif]

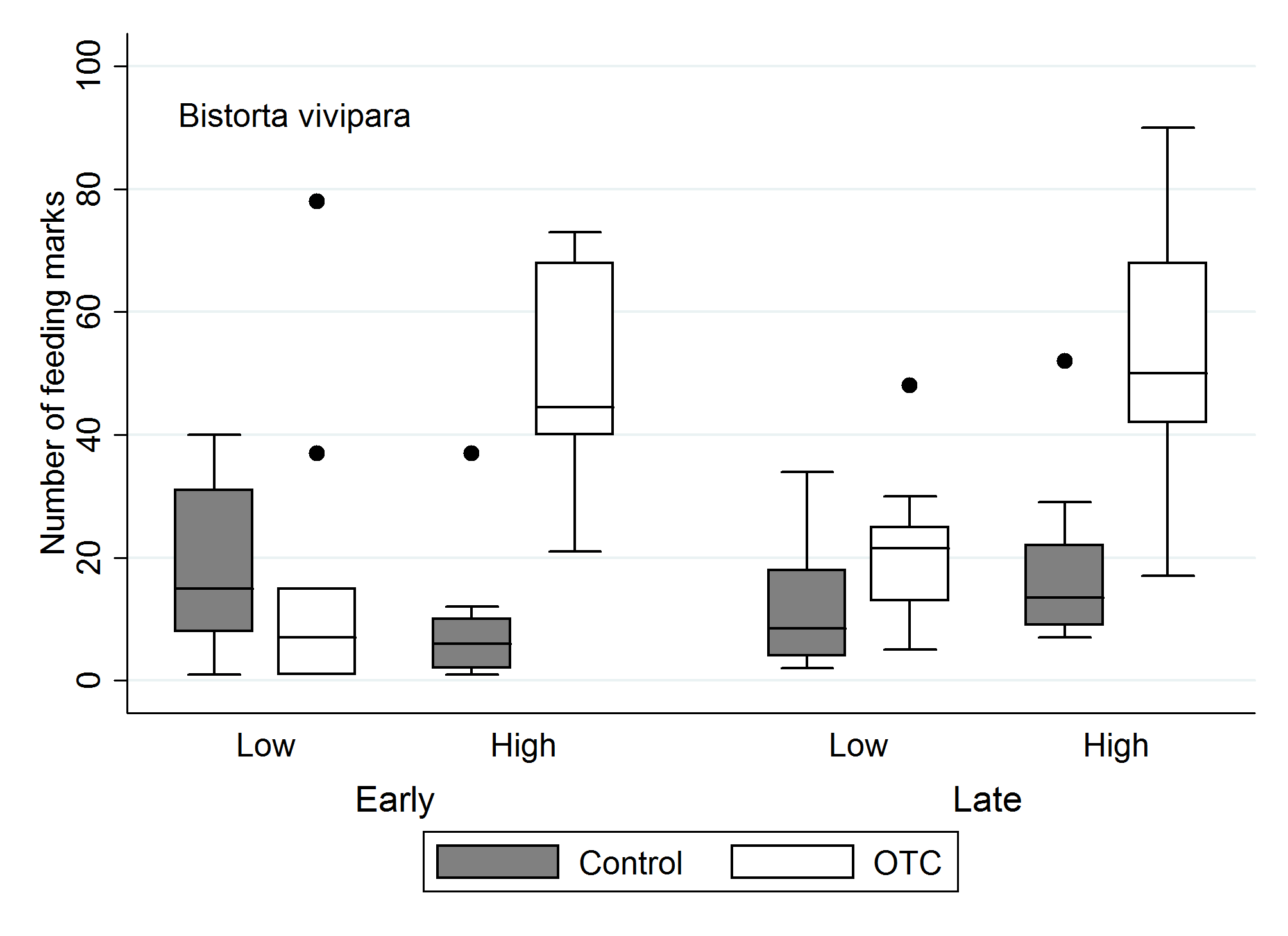

Supplement: Supplementary file 2 — Figure S2. Number of feeding marks on B. vivipara in control and OTC plots at a high and a low elevation site recorded in early and late summer in an alpine Dryas heath at Finse, Norway. [file ECE3-6-6955-s002.tif]
