# Supplementary material for: Experimental warming increases herbivory by leaf‐chewing insects in an alpine plant community
Source: Ecol Evol. 2016 Sep 7;6(19):6955–62. doi: 10.1002/ece3.2398 (PMC5513215; doi:10.1002/ece3.2398)
Supplement: Supplementary file 3 — Table S1. ANCOVA results with number of feeding marks on D. octopetala and B. vivipara as response variables explained by site, treatment and the percentage cover of these species within the plots. [file ECE3-6-6955-s003.docx]

**TABLE S1**

ANCOVA results with number of feeding marks on *D. octopetala* and *B. vivipara* as response variables explained by site, treatment and the percentage cover of these species within the plots. Significant (p < 0.05) effects are in bold. All response variables were log-transformed in the analysis.

|  | Early summer | | |  | Late summer | |
| --- | --- | --- | --- | --- | --- | --- |
| *Dryas octopetala* | Df | t-ratio | p-value |  | t-ratio | p-value |
| Site (Low-high) | 1 | **2.21** | **0.0344** |  | **4.43** | **0.0001** |
| Treatment (OTC-Control) | 1 | -1.74 | 0.0917 |  | **-3.54** | **0.0013** |
| Site*Treatment | 1 | 0.02 | 0.9864 |  | **-2.04** | **0.0502** |
| % *Dryas* | 1 | **2.59** | **0.0145** |  | **3.65** | **0.0010** |
| % *Dryas**Treatment | 1 | -0.56 | 0.5819 |  | -1.32 | 0.1957 |
|  |  | *n=37, R^2^=0.23* | |  | *n=37, R^2^=0.50* | |
| *Bistorta vivipara* |  |  |  |  |  |  |
| Site (Low-high) | 1 | **-3.45** | **0.0016** |  | **-4.37** | **0.0001** |
| Treatment (OTC-Control) | 1 | **-2.88** | **0.0069** |  | **-4.05** | **0.0003** |
| Site*Treatment | 1 | **4.03** | **0.0003** |  | 1.35 | 0.1850 |
| % *Bistorta* | 1 | **4.32** | **0.0001** |  | **2.19** | **0.0353** |
| % *Bistorta**Treatment | 1 | 0.87 | 0.3893 |  | -0.03 | 0.9781 |
|  |  | *n=39, R^2^=0.65* | |  | *n=40, R^2^=0.57* | |
